# A protocol for a systematic review of standardised tools used in perinatal death review programmes

**DOI:** 10.12688/hrbopenres.13574.2

**Published:** 2023-08-24

**Authors:** Emily O'Connor, Richard Greene, Keelin O'Donoghue, Sara Leitao

**Affiliations:** 1 INFANT Research Centre, University College Cork, Cork, Ireland; 2 National Perinatal Epidemiology Centre, University College Cork, Cork, Ireland; 3 Pregnancy Loss Research Group, Department of Obstetrics & Gynaecology, University College Cork, Cork, Ireland

**Keywords:** Perinatal mortality, stillbirth, neonatal death, review tool, surveillance and response, protocol

## Abstract

**Introduction:** Perinatal mortality encompasses stillbirths and early neonatal deaths. A perinatal death surveillance and response cycle has been recommended by the World Health Organization for use in the review of perinatal deaths. The main components of the cycle include identifying and reporting perinatal deaths, and reviewing the deaths, including potentially modifiable factors, in order to measure and improve quality of care provided to women and infants. There is no consensus on the best way to design, implement and conduct perinatal death reviews. This systematic review aims to identify standardised tools that are used to review perinatal deaths.

**Objectives:** The primary aim of this protocol is to describe methodology for a systematic search of the literature to identify standardised tools that are used to review perinatal deaths in upper-middle to high-income countries. Review tools may include standardised checklists, forms, frameworks or other structured documents used to review perinatal deaths. Review tools will be appraised to see if they incorporate the identification of modifiable factors in perinatal deaths and establish recommendations for improvements to quality of care provided.

**Methods:** A systematic review of the literature will be performed to identify peer-reviewed publications and grey literature describing the use of perinatal mortality review tools without date restrictions. The eligibility of review tools for inclusion will be based on inclusion and exclusion criteria applied to the SPIDER framework. Data will be extracted based on the structure and content of included review tools, and the tools will be appraised using the Appraisal of Guidelines Research and Evaluation Health Systems (AGREE-HS) instrument.

**Conclusion**: This systematic review protocol for identifying and appraising standardised perinatal mortality review tools may help to establish the optimal way to structure a standardised review process for perinatal mortality in middle- to high-income countries.

**PROSPERO registration**: CRD42022326877

## Introduction

“The day of birth is potentially the most dangerous day for both mothers and their babies” according to the World Health Organisation (WHO) ^1^. While significant reductions have been made in neonatal mortality in the last two decades, there are still an estimated 2.7 million neonatal deaths every year. It is worth noting that there are also an estimated 2.6 million stillbirths every year, a fact that does not feature as prominently in the Sustainable Development Goals (SDG) published by the United Nations in 2016 ^2^. Although the SDG 3.2 includes a target reduction in neonatal mortality to 12/1,000 livebirths by 2030, there is no clear reference to a reduction of stillbirth rates in this document ^2^.

Perinatal mortality includes stillbirths (both antepartum and intrapartum stillbirths) and early neonatal deaths (death of a live born infant occurring within the first seven days of life). The perinatal mortality rate (PMR) of a country is a key indicator of the quality of maternity services available in that country ^3, 4^. In 2019, a stillbirth rate of 3.0/1000 livebirths and a neonatal death rate of 2.9/1000 livebirths was reported ^5, 6^. Perinatal death surveillance is a powerful approach towards monitoring and improving healthcare services ^4^. A perinatal death is considered in many countries to be a sentinel event, which is defined in the United States (US) by the Joint Commission as a patient safety event that results in death, permanent harm, or severe temporary harm ^7^. In an Irish context, a perinatal death is considered a serious reportable event, which is defined as a subset of incidents which are either serious or that should not occur if the available preventative measures have been effectively implemented by healthcare providers ^8^. In recent years, there has been a greater focus on auditing and reviewing the circumstances surrounding the perinatal death with particular emphasis on the role of system factors in the death ^1, 8– 10^.

In support of this, and to emphasise the importance of multi-factor review of perinatal deaths, the WHO published guidance on the establishment of maternal and perinatal death surveillance and response (MPDSR) committees at a local level in 2021 ^9^. The value of comprehensive review of perinatal deaths has already been established in several high-income countries around the world ^10, 11^. This includes the United Kingdom (UK), where the perinatal mortality rate (PMR) has reduced by 18% from 2012–2018 with the introduction of several targeted measures, one of which is a perinatal mortality review tool (PMRT) ^12^. Review tools, as part of this process, allow for a more streamlined approach to identification of risk factors and care-related issues. Similarly, the Netherlands established the Foundation National Perinatal Audit (PAN) programme in 2010 (now known as PERINED), which standardised the perinatal death review process, and helped to reduce their PMR by 18% during the years 2010 to 2015 ^13^.

While it is extremely important to capture data on number and causes of perinatal deaths, it is also of vital importance that review of perinatal deaths encompasses the identification of modifiable factors in those deaths. Modifiable factors are defined by the WHO as a factor “that may have prevented the death had a different course of action been taken” ^1, 9^. Identification of remediable factors forms part of the WHO’s MPDSR cycle. It is only through reviewing quality of care that quality improvements and changes to clinical care can be enacted, thereby completing the cycle ^1^. The importance of evaluating the quality of care through structured review has been highlighted repeatedly by MBRRACE-UK, whose confidential enquiry and national audit is often viewed internationally as a gold standard ^10^. The quality of institutional review was more recently emphasised in the Ockenden Report, which reviewed the maternity services at a hospital group in the UK ^14^. Recommendations from this report included strengthened accountability amongst senior maternity staff, timely implementation of changes in practice following review of care and improving family engagement in investigations.

There is no international consensus on the optimum way to conduct perinatal death reviews that factor in all of the above-mentioned aspects of the review cycle. A 2020 Cochrane systematic review of death audits for reducing maternal, perinatal and child mortality concluded that more research is required in order to identify how death reviews should be designed and implemented in order to achieve maximum effectiveness in different contexts globally ^3^.

While there are some support materials for maternal and perinatal death review included in the MPDSR guidance document published by the WHO in 2021, many of the examples given focus largely on maternal death reviews and surveillance ^9^. Included information on perinatal death reviews, for example the sample stillbirth and neonatal death case review form, provides limited information and lacks the detail required to identify risk and contributory factors. which is possibly not as suited to countries who may have access to larger dataset and information on each perinatal death.. There is a need to identify what standardised tools (if any) are in use internationally for the review of perinatal deaths that may be more suitable for use in high-income, resource-rich settings.

The evidence for appraisal or comparison of review tools for perinatal deaths is lacking in the literature. This is particularly the case for review tools in use in high-income settings that incorporate the development and implementation of recommendations for clinical practice and governance. In addition, this analysis may aid policy-makers and stakeholders who seek to implement a standardised review tool to evaluate perinatal deaths in their own institution or country.

## Primary aim

The primary aim of this systematic review is to examine standardised tools used to review perinatal deaths.

## Objectives:

- Identify tools or other standardised checklists, forms, frameworks or other documents that are currently in use or have been piloted for use in reviewing perinatal deaths.
- Describe the structure of identified perinatal review tools and if they generate data or information on modifiable factors contributing to perinatal deaths.
- Assess if identified facilitators and barriers to the implementation of standardised perinatal mortality review tool.
- Examine evidence of validation or accreditation of the tool identified.

As part of this systematic review, we will also study the development of recommendations for clinical care generated as part of the use of standardised tools to review perinatal deaths and examine the evidence that employing these tools may contribute towards a reduction of perinatal mortality rates at institution, region or country-level.

## Protocol and registration

This protocol has been registered in the International Prospective Register of Systematic Reviews (PROSPERO) database (protocol number: CRD42022326877) and is reported in accordance with the reporting guidance provided in the Preferred Reporting Items for Systematic review and Meta-Analysis – Protocols (PRISMA-P) 2015 ^15^; the completed checklist is available as part of the extended data for this project.

## Eligibility criteria

The eligibility of review tools will be based on inclusion and exclusion criteria applied to the SPIDER framework (S: sample, PI: population of interest, D: study design, E: evaluation, R: research type), developed by Cooke *et al.* and outlined in Table 1 ^16^.

**Table 1.** SPIDER framework for eligibility criteria.

SPIDER Framework	Eligibility Criteria
**S: Sample**	– Review of perinatal deaths (stillbirths and/or early neonatal deaths) occurring in maternity units or hospitals – Standardised review tools used, in use or piloted for use at local, regional or national level – Upper-middle-income to high-income countries as defined by the World Bank 2022 (N=135) ^18^
**PI: Phenomenon of** ** Interest**	– Perinatal mortality: stillbirths (antepartum and intrapartum) and early neonatal deaths – Definition according to each country
**D: Study Design**	– Any study, report or other publication detailing the current use or trial of use of a standardised review tool to review individual perinatal deaths or perinatal mortality as a whole – No language restriction – No date restriction
**E: Evaluation**	– Structure, content and format of perinatal mortality review tools – Standardisation or validation of the review tool – Development of recommendations based on identified remediable factors based on the review of perinatal deaths – Evidence of reduction in perinatal mortality rate (PMR) in the relevant institution, region or country – Facilitators and barriers encountered in the implementation of the perinatal mortality review process
**R: Research type**	– All relevant study types involving the use of a standardised tool to review perinatal deaths will be included, including both quantitative and qualitative studies – Grey literature including publications from national or international scientific societies, professional colleges, charitable organisations, and government organisations

The definition of stillbirth varies widely internationally. The WHO identifies the following definition for stillbirth: “death before birth, among fetuses that are, by order of priority, of at least 1000g birthweight, and/or at least 28 weeks gestation, and at least 35cm long.” ^9^ Some countries use lower thresholds for both gestational age and weight at birth to define stillbirth (for example, a fetus that has reached greater than or equal to 24 weeks’ gestation or weighing greater than 500g at birth) ^17^. As the term “perinatal mortality” is generally accepted to encompass stillbirths (antepartum and intrapartum) and early neonatal deaths, this definition also varies internationally. For the purposes of this systematic review, a country’s own definition of perinatal mortality, stillbirth and early neonatal death will be used when searching for tools used to review perinatal deaths.

Literature focused on tools form upper-middle-income to high-income countries to allow better comparability and ensure that the tools apply to settings with somewhat similar characteristics and level of resources.

In addition, the search will not be restricted by language, date of publication or type of study.

## Information sources

The identification of relevant perinatal mortality review tools will encompass a multi-tiered approach. Systematic bibliographic database searching will identify review tools that are in use or have been piloted for use at a local, regional or national level by individual hospitals, hospital groups or committees. This includes identifying review tools published in journals, through professional medical associations and organisations, or identified through searches of grey literature.

A systematic literature search will be performed to identify publications describing the use of perinatal mortality review tools using the following databases: PubMed, EMBASE, Cumulative Index to Nursing and Allied Health Literature (CINAHL), Web of Science and OpenGrey. Websites of international scientific institutes, organisations & professional societies, listed in Table 2, will also be searched. Other professional organisations will be added to this table if applicable as the search progresses.

**Table 2.** Scientific institutes, organisations & professional societies.

Scientific Institutes, Organisations & Professional societies	Country/Region
National Perinatal Epidemiology Centre (NPEC)	Ireland
Mothers and Babies: Reducing Risk through Audits and Confidential Enquires across the UK (MBRRACE-UK)	U.K
French Society of Neonatology	France
Dutch Society of Obstetrics and Gynecology	The Netherlands
German Society of Perinatal Medicine	Germany
European Society of Human Reproduction and Embryology (ESHRE)	Europe
European Association of Obstetricians and Gynecologists	Europe
European Association of Perinatal Medicine	Europe
European Board and College of Obstetrics and Gynecology (EBCOG)	Europe
Union of European Neonatal and Perinatal Societies (UENPS)	Europe
Perinatal Society of Australia and New Zealand (PSANZ)	Australia & New Zealand
National Perinatal Association	U.S.A.
Canadian Perinatal Network	Canada
Society for Maternal-Fetal Medicine (SMFM)	International
World Association of Perinatal Medicine (WAPM)	International
The International Federation of Gynaecology and Obstetrics (FIGO)	International

## Search strategy

A search strategy will be developed by the study investigators. The search strategy will include suitable keywords relating to perinatal mortality reviews. The terms will be followed by the truncation symbols * and will be refined using Boolean operators such as AND/OR. The initial search will be limited to screening titles and abstracts rather than full bibliographic records. The search strategy will be similar for all databases (with the same search terms and combinations) with exceptions for where a a specific database criteria requires a tailored approach. An example of search strategies developed for use in scientific databases is included in Supplementary File 1. The search strategy will be piloted before the final searches are executed.

## Study selection

For the bibliographic database search, two reviewers will screen titles and abstracts against the inclusion and exclusion criteria outlined below.

Inclusion criteria:

- Perinatal mortality review tools from upper-middle- to high-income countries as classified by the World Bank Country and Lending Groups 2022 (N=135) ^18^
- Review tools for perinatal deaths currently in use or review tools that have been piloted for use in individual maternity units or hospitals, or in hospital groups
- Review tools for perinatal deaths in use at local, regional or national level
- Review tools for perinatal deaths that include a recommendation section for changes to clinical practice, guidelines, and/or governance
- Tools that are used in the review of individual perinatal deaths or tools that are used for reviewing a grouping of perinatal deaths (e.g. intrapartum stillbirths or stillbirths caused by placental abruption)
- Review tools available in any language
- No date restrictions

Exclusion criteria:

- Tools previously used to review perinatal deaths that are no longer in use or have been decommissioned or replaced
- Lower-middle- and low-income countries as classified by the World Bank Country and Lending Groups 2022
- Publication does not include the review or audit tool nor enough relevant information on this in the published material, or the review tool cannot be accessed by other means (e.g. direct contact with the study authors, or the governing group)

For screening of websites, two independent reviewers will screen the websites for the scientific institutes, organisations & professional societies outlined above. The titles and abstracts (where applicable) will be reviewed against the inclusion/exclusion criteria outlined above. The methodology for this website screening follows the process previously described by Hennessy *et al.* (2021) in their research ^19^.

Results from both the scientific databases and the website screening will be imported using Rayyan software ^20^ and duplicates will be removed. Title and abstract (or summary) screening will be carried out in this software using the blinding feature during the review period. When abstracts are not available, the summary of the document will be used. If summaries or preliminary information on the document is not available, a brief screening of the full record will be carried out. The SPIDER framework will be applied broadly during the initial screening to ensure relevant perinatal mortality review tools are not excluded. Full text articles of publications identified through screening of titles and abstracts will be retrieved and assessed independently for eligibility by the two reviewers. Disagreement between the two reviewers regarding the eligibility of any study will be resolved following discussion and consensus. If consensus on eligibility is not reached, the opinion of a third reviewer will be sought.

A PRISMA flow diagram will be used to illustrate the search process. This diagram will map out the process for review tool selection and the number of records identified at each stage based on the inclusion and exclusion criteria.

## Data collection

Once the review tools for inclusion have been obtained, all associated supplementary documents will be retrieved by the reviewer (EOC) prior to data extraction and quality assessment. If links to these documents are not provided within the article, website or other source, EOC will conduct a search to locate them. All supplementary documents will be verified by a second reviewer to ensure completeness and correct document pairing.

## Data extraction

Data extraction will be completed by one reviewer (EOC) and will be independently verified by a second reviewer for accuracy and completeness. A standardised, pre-piloted form in Microsoft Excel will be used to extract data from the identified perinatal mortality review tools for assessment of quality and data synthesis. Discrepancies will be resolved through discussion and consensus. If consensus cannot be reached, the opinion of a third reviewer will be sought. Any missing information from the review tools will be recorded as ‘not described’ in the data extraction form.

The following information will be extracted from identified perinatal mortality review tools:

- General Information
  - Title
  - Author & year of publication
  - Language
  - Developing/publishing organisation and/or authors/ funding
  - Country/countries of publication
  - Version

- Description of document provided by the authors (e.g. guideline, review tool, audit tool)
- Composition of tool development group, if applicable
- Evidence of peer review or validation
- Target user of the review tool
- Type of perinatal death reviewed by the tool (e.g. all perinatal deaths, intrapartum stillbirths only, or early neonatal deaths only)
- Document structure (including subsections, type of data collected, length of document)
- Development process (evidence based and/or consensus-based)
  - If a recommendation development section is included, data extraction will include the structure of the recommendation section
  - Appraisal of recommendations based on SMART principles (Specific, Measurable, Achievable, Relevant, Time-bound) ^21^

- Framework used for the development of recommendations, e.g. the Yorkshire framework ^22^
- Validity period, if specified

## Quality assessment

The quality of the included review tools will be appraised using the Appraisal of Guidelines Research and Evaluation Health Systems (AGREE-HS) tool ^23^. This tool was developed by the AGREE research team to systematically appraise health systems guidance (or guideline) documents produced by countries, governing bodies or committees at national, regional or local level. Two reviewers will independently conduct the quality assessment of the review tools using AGREE-HS. This tool focuses on five key domains that form part of the development of health systems guidance:

1. Topic
2. Participants
3. Methods
4. Recommendations
5. Implementability

Each domain is applied to the guidance document (in this case, the perinatal mortality review tool) and scored using a 7-point Likert scale, with scores ranging from 1 (lowest quality) to 7 (highest quality). A final score for the overall quality of the review tool will be calculated by totalling the individual scores for the five domains. If consensus cannot be research during appraisal (i.e., if individual scores from each reviewer differ more than 2 points), a third reviewer (KOD) will assist with the appraisal process. The final scores will be used to help interpret the guidance documents being appraised by identifying review tools of higher and/or lower quality. The quality appraisal outcomes will not affect the literature that will be included in the data synthesis.

## Data synthesis

Data synthesis will involve a descriptive approach to appraisal and examination of perinatal mortality review tools. The appraisal will be conducted using the AGREE-HS tool, as described above. There will be a narrative description of the data extracted from the perinatal mortality review tools, as well as a specific focus on those tools that include a section on the development of recommendations based on the review findings (if applicable). 

## Conclusion

The aim of this systematic review is to identify and appraise standardised review tools that are currently in use or have been piloted for use in reviewing perinatal deaths. Consistent review of the modifiable factors around perinatal deaths and implementation of recommendations developed from the review may help to prevent future perinatal deaths and reduce a country’s PMR. There is a need to identify what standardised review tools are being used to review perinatal deaths, particularly in a high-income, resource-rich setting and appraise and compare the tools. The findings from this systematic review may help in the implementation of a targeted perinatal death review program.

## Dissemination

We plan for this systematic review of perinatal mortality review tools to be disseminated through peer-reviewed publication and presentation at relevant professional and scientific events.

## Data availability

### Underlying data

No data are associated with this article.

### Extended data

Open Science Framework: A protocol for a systematic review of standardised tools used in perinatal death review programmes, https://doi.org/10.17605/OSF.IO/PVKX7 ^24^.

This project contains the following extended data:

- Supplementary File 1 Sample Search Strategy.pdf

### Reporting guidelines

Open Science Framework: PRISMA-P checklist for “ *A protocol for a systematic review of standardised tools used in perinatal death review programmes*”, https://doi.org/10.17605/OSF.IO/PVKX7.

Data are available under the terms of the Creative Commons Zero "No rights reserved" data waiver (CC0 1.0 Public domain dedication).
