# Supplementary material for: A protocol for a systematic review of standardised tools used in perinatal death review programmes
Source: HRB Open Res. 2023 Aug 24;5:52. Originally published 2022 Jul 13. [Version 2] doi: 10.12688/hrbopenres.13574.2 (PMC10518843; doi:10.12688/hrbopenres.13574.2)
Supplement: Peer review history

# Reviewer report: Reviewer response for version 2

**DOI:** 10.21956/hrbopenres.15093.r35866

**Published:** 2023-09-11

**Authors:** Collette N Ncube, Ruby Barnard-Mayers

**Affiliations:** 1 Boston University, Boston, Massachusetts, USA

No further comments

Is the study design appropriate for the research question?

Yes

Is the rationale for, and objectives of, the study clearly described?

Partly

Are sufficient details of the methods provided to allow replication by others?

Partly

Are the datasets clearly presented in a useable and accessible format?

Not applicable

Reviewer Expertise:

Epidemiology, Maternal and Child Health

We confirm that we have read this submission and believe that we have an appropriate level of expertise to confirm that it is of an acceptable scientific standard.

# Reviewer report: Reviewer response for version 2

**DOI:** 10.21956/hrbopenres.15093.r35867

**Published:** 2023-09-08

**Authors:** Rachidatou Compaoré

**Affiliations:** 1 Institut de Recherche en Sciences de la Santé, Ouagadougou, Burkina Faso

I assessed the authors' revision, and I would like to confirm through this email that I am happy with the changes and have no additional comments.

Is the study design appropriate for the research question?

Yes

Is the rationale for, and objectives of, the study clearly described?

Partly

Are sufficient details of the methods provided to allow replication by others?

Partly

Are the datasets clearly presented in a useable and accessible format?

Not applicable

Reviewer Expertise:

Public health, sexual and reproductive health, quality of care, health policy and systems research, process evaluation

I confirm that I have read this submission and believe that I have an appropriate level of expertise to confirm that it is of an acceptable scientific standard.

# Reviewer report: Reviewer response for version 1

**DOI:** 10.21956/hrbopenres.14820.r34967

**Published:** 2023-08-01

**Authors:** Collette N Ncube, Ruby Barnard-Mayers

**Affiliations:** 1 Boston University, Boston, Massachusetts, USA

Thank you for the opportunity to review this study protocol aimed at identifying standardised tools used in perinatal death review programmes in high- and middle-income countries. In addition to summarizing the tools identified, the authors are particularly interested in describing facilitators and barriers to implementation of these tools, and determining whether the tools are structured to support the development of recommendations for change to clinical practice based upon identified modifiable factors contributory to perinatal deaths.

**Introduction**

*1 ^st^ paragraph*: The authors allude to the existence of a Sustainable Development Goal related to neonatal death (SDG 3.2 which includes a target reduction in neonatal mortality rates) and the absence of an SDG target for stillbirth. However, this is not mentioned explicitly. We would recommend that the authors provide more clarity to their statement.

*3 ^rd^ paragraph*: This paragraph could be strengthened by the addition of information regarding the ways in which the process of perinatal death review has led to the reduction in PMRs in the identified countries. It can be inferred from the manuscript that clinical policy recommendations may result from this exercise, and that perhaps it is the goal of the authors to determine whether that is the case by conducting this review. It would be helpful to the reader to know the hypothesized or empirically identified mechanisms by which perinatal death review contributes to reductions in perinatal deaths.

*6 ^th^ paragraph*: Additional information is required regarding the inadequacy of the MPDSR perinatal death review for application to upper-middle- and high-income countries. The authors indicate that the MPDSR perinatal death review requires less information for the conduct of the review. This alone does not make it “unsuitable for use in high-income, resource-rich settings” as we could assume that information would be readily available in resource-rich settings. The authors might consider excluding the comparison between low-income and high-income countries and focus instead on the value such a review could provide to upper-middle- and high-income countries.

*7 ^th^ paragraph*: The authors mention the Republic of Ireland as the target population for policy change following this review, however the introduction is not focused on this country. It might be better to exclude this statement and bring it up for discussion in the following paper discussing results from the review.

**Primary Aim**

 No comments.

**Objectives**

 No comments.

**Protocol and registration**

 No comments.

**Eligibility criteria**

 Please include the rationale for including only high- and middle-income countries.

 We recommend moving the definitions of stillbirth given in this section to the introduction.

**Information sources**

 No comments.

**Search strategy**

 The SPIDER search tool proposed by Cooke, Smith & Booth (2012) is one of a number of variations to the PICO approach for formulation a search strategy in a literature review of qualitative studies. We think rationale is required for choosing the SPIDER search tool/framework to define the literature review eligibility criteria and, assumedly, the search strategy that will be developed for this systematic literature review. Among the number of variations to the PICO approach, the PICOS tool appears to be the recommended tool for literature reviews of qualitative research (Methley *et al. *(2014) ^1^ and Booth (2016) ^2^).

 The protocol could benefit from some clarity regarding the number of reviewers who will be involved in title and abstract review of citations from electronic databases (such as PubMed and Embase); specific mention of the use of Rayyan for title and abstract review of citations from electronic databases, if applicable; and any blinding during review of titles and abstracts. The process by which the websites will be screened lacks the level of detail required to replicate the review. We would suggest that the authors describe this aspect of the review in more detail, referencing any prior work describing the process for such a review if any exists. The authors mention that the “Results will be imported using Rayyan software.” Some specificity would be helpful regarding the results referenced here; particularly, whether this includes websites. Since websites are less likely to have titles and abstracts as do peer-reviewed journal articles, it is unclear how their content will be evaluated alongside the citations within Rayyan.

**Quality Assessment**

 The authors describe the scoring system for the AGREE-HS quality assessment tool, which would result in a total score for each review tool identified via this systematic literature review process ranging from 10-70 (score of 1 * 5 items * 2 reviewers; score of 7 * 5 items * 2 reviewers). We would recommend that the authors include an explanation of what consensus looks like in this case where the outcome is a total score across the two reviewers (i.e., do the individual scores have to be identical or can they vary by 1 or 2 points).

**References**

 The URL link for Reference 6 link is invalid.

**Supplementary files**

 Inclusion of the sample search strategy for the CINAHL database permits reproducibility of the literature review. It would be helpful to include a sample search strategy for all databases to be searched in this review.

Is the study design appropriate for the research question?

Yes

Is the rationale for, and objectives of, the study clearly described?

Partly

Are sufficient details of the methods provided to allow replication by others?

Partly

Are the datasets clearly presented in a useable and accessible format?

Not applicable

Reviewer Expertise:

Epidemiology, Maternal and Child Health

We confirm that we have read this submission and believe that we have an appropriate level of expertise to confirm that it is of an acceptable scientific standard, however we have significant reservations, as outlined above.

## References

[1] : PICO, PICOS and SPIDER: a comparison study of specificity and sensitivity in three search tools for qualitative systematic reviews. *BMC Health Serv Res* .2014;14: 10.1186/s12913-014-0579-0 579 10.1186/s12913-014-0579-0 25413154PMC4310146

[2] : Searching for qualitative research for inclusion in systematic reviews: a structured methodological review. *Syst Rev* .2016;5: 10.1186/s13643-016-0249-x 74 10.1186/s13643-016-0249-x 27145932PMC4855695

# Response

**Published:** 2023-08-18

**Authors:** Sara Leitao

**Affiliations:** National Perinatal Epi. Centre, University College Cork, Cork, County Cork, Ireland

Thank you for your review of this Systematic Review Protocol.

 we hope to have addressed all the comments and suggestions in the new draft and we outline, below, an itemized response and clarification on each of the comments by these reviewers.

**Introduction**

*1 ^st^ paragraph*: The authors allude to the existence of a Sustainable Development Goal related to neonatal death (SDG 3.2 which includes a target reduction in neonatal mortality rates) and the absence of an SDG target for stillbirth. **However, this is not mentioned explicitly. We would recommend that the authors provide more clarity to their statement**.

**Response**

 Thank you for highlighting this, further clarity on SDG3.2 and absence of a clear reference to SB has been included in paragraph 1.

*3 ^rd^ paragraph*: This paragraph could be strengthened by **the addition of information regarding the ways in which the process of perinatal death review has led to the reduction in PMRs in the identified countries.** It can be inferred from the manuscript that clinical policy recommendations may result from this exercise, and that perhaps it is the goal of the authors to determine whether that is the case by conducting this review. It would be helpful to the reader to know the hypothesized or empirically identified mechanisms by which perinatal death review contributes to reductions in perinatal deaths.

**Response**

 This has been clarified further in paragraph 3.

*6 ^th^ paragraph*: **Additional information is required regarding the inadequacy of the MPDSR perinatal death review for application to upper-middle- and high-income countries.** The authors indicate that the MPDSR perinatal death review requires less information for the conduct of the review. This alone does not make it “unsuitable for use in high-income, resource-rich settings” as we could assume that information would be readily available in resource-rich settings. The authors might consider excluding the comparison between low-income and high-income countries and focus instead on the value such a review could provide to upper-middle- and high-income countries.

**Response**

 Thank you for highlighting this. We have rephrased this paragraph to clarify the argument being made.

*7 ^th^ paragraph*: The **authors mention the Republic of Ireland as the target population for policy change following this review, however the introduction is not focused on this country**. It might be better to exclude this statement and bring it up for discussion in the following paper discussing results from the review.

**Response**

 The reference to Republic of Ireland as target population has now been removed.

**Eligibility criteria**

 Please include the rationale for including only high- and middle-income countries.

**Response**

 This has now been included in the 3rd paragraph of the “Eligibility criteria” section.

**Eligibility criteria**

 We recommend moving the definitions of stillbirth given in this section to the introduction.

**Response**

 We appreciate the suggestion and have given thoughtful consideration to this. The definition of stillbirth is placed in this section to provide clearer context on the criteria that will be used within the systematic review and hence we believe it is of greater value in the current location.

**Search strategy**

 The SPIDER search tool proposed by Cooke, Smith & Booth (2012) is one of a number of variations to the PICO approach for formulation a search strategy in a literature review of qualitative studies. We think rationale is required for choosing the SPIDER search tool/framework to define the literature review eligibility criteria and, assumedly, the search strategy that will be developed for this systematic literature review. Among the number of variations to the PICO approach, the PICOS tool appears to be the recommended tool for literature reviews of qualitative research (Methley *et al. *(2014) ^1^ and Booth (2016) ^2^ ).

**Response**

 SPIDER was considered more relevant for this SR as it does not look at interventions specifically, not in a changed/comparison of outcomes. This review looks at the various tools available to review Perinatal Deaths (the Phenomenon of Interest) and hence a tool focussed on outcomes would not be the most applicable. As per CASP: "PICO mostly focuses on intervention (or therapy) clinical questions. It can be less suitable for other question types (such as qualitative research) as it doesn’t account for some complexities like considering feasibility, context, and sociocultural acceptability " ( https://casp-uk.net/pico-framework/ 02 Aug 23)

 The protocol could benefit from some **clarity regarding the number of reviewers who will be involved in title and abstract review of citations from electronic databases** (such as PubMed and Embase); **specific mention of the use of Rayyan for title and abstract review of citations from electronic databases, if applicable; and any blinding during review of titles and abstracts**. The **process by which the websites will be screened lacks the level of detail required to replicate the review**. We would suggest that the authors describe this aspect of the review in more detail, referencing any prior work describing the process for such a review if any exists. The authors mention that the **“Results will be imported using Rayyan software.” Some specificity would be helpful regarding the results referenced here; particularly, whether this includes websites. Since websites are less likely to have titles and abstracts as do peer-reviewed journal articles, it is unclear how their content will be evaluated alongside the citations within Rayyan**.

**Response**

 Further detail on the items listed above has been included in paragraph 4 and 5 of the “study Selection” section.

**Quality Assessment**

 The authors describe the scoring system for the AGREE-HS quality assessment tool, which would result in a total score for each review tool identified via this systematic literature review process ranging from 10-70 (score of 1 * 5 items * 2 reviewers; score of 7 * 5 items * 2 reviewers). **We would recommend that the authors include an explanation of what consensus looks like in this case** where the outcome is a total score across the two reviewers (i.e., do the individual scores have to be identical or can they vary by 1 or 2 points).

**Response**

 Thank you for highlighting the need for this further clarification in the manuscript, this has now been included.

**References**

 The URL link for Reference 6 link is invalid.

**Response**

 This has now been corrected and the correct URL provided.

**Supplementary files**

 Inclusion of the sample search strategy for the CINAHL database permits reproducibility of the literature review. It would be helpful to **include a sample search strategy for all databases to be searched in this review.**

**Response**

 This has now been included as supplementary file.

# Reviewer report: Reviewer response for version 1

**DOI:** 10.21956/hrbopenres.14820.r33933

**Published:** 2023-07-04

**Authors:** Rachidatou Compaoré

**Affiliations:** 1 Institut de Recherche en Sciences de la Santé, Ouagadougou, Burkina Faso

This article is a systematic review protocol for a study on standardised tools used in perinatal death review programmes. The study aims to identify the best practices for reviewing perinatal deaths and improving the quality of care provided to women and infants. The review will be conducted using a comprehensive search strategy and will include studies that evaluate the use of standardised tools in perinatal death review programmes in high income countries.

 The rationale for, and objectives of, the study clearly described, The rationale is well presented with a justification to conduct the study. It would be interesting to precise the perinatal rates in high income countries.

 The authors were also careful to specify their general and specific objectives for this study. However, the general objective is not well formulated, containing three verbs for a single objective. There are too many specific objectives: some of the specific objectives could be grouped together. In addition, the authors could try to present the specific objectives in a more organised way, for example everything concerning the structure of the tools should be grouped together, and everything concerning the actions taken following the generation of information grouped together too. Some specific objectives go beyond the general objective, which seems more descriptive, whereas some specific objectives refer to actions taken to correct situations leading to deaths and improve mortality rates. 

 The authors presented their methods in a structured manner. It would be necessary to describe the databases that the authors intend to screen and also add the search strategies for each of the database.

Is the study design appropriate for the research question?

Yes

Is the rationale for, and objectives of, the study clearly described?

Partly

Are sufficient details of the methods provided to allow replication by others?

Partly

Are the datasets clearly presented in a useable and accessible format?

Not applicable

Reviewer Expertise:

Public health, sexual and reproductive health, quality of care, health policy and systems research, process evaluation

I confirm that I have read this submission and believe that I have an appropriate level of expertise to confirm that it is of an acceptable scientific standard, however I have significant reservations, as outlined above.

# Response

**Published:** 2023-08-18

**Authors:** Sara Leitao

**Affiliations:** National Perinatal Epi. Centre, University College Cork, Cork, County Cork, Ireland

Many thanks for the review and comments on our manuscript

 As per your suggestion, a reference to the perinatal rates in high income countries has now been included in the second paragraph of the introduction.

 We have also rephrased the primary aim and re-structured the objectives as per reviewer's suggestion.

 In relation to the suggestion to describe the databases to use and add the search strategies for each of the database: the databases to use in the literature search are listed in the second paragraph of the section “Information sources”. Information on search strategies and for each database has been updated as outlined in the section “Search strategy”. Table 3 has also been replaced with a supplementary table with a more detailed example of the search strategies developed.
